# In-network generalized trustworthy data collection for event detection in cyber-physical systems

**DOI:** 10.7717/peerj-cs.504

**Published:** 2021-05-04

**Authors:** Hafiz Ur Rahman, Guojun Wang, Md Zakirul Alam Bhuiyan, Jianer Chen

**Affiliations:** 1 School of Computer Science, Guangzhou University, Guangzhou, Guangdong Province, China; 2 Department of Computer and Information Sciences, Fordham University, New York, NY, United States

**Keywords:** Cyber-physical system, Data quality, Event monitoring, Data trustworthiness, Data dependability, Security and privacy, Data collection, Fire detection

## Abstract

Sensors in Cyber-Physical Systems (CPS) are typically used to collect various aspects of the region of interest and transmit the data towards upstream nodes for further processing. However, data collection in CPS is often unreliable due to severe resource constraints (e.g., bandwidth and energy), environmental impacts (e.g., equipment faults and noises), and security concerns. Besides, detecting an event through the aggregation in CPS can be intricate and untrustworthy if the sensor's data is not validated during data acquisition, before transmission, and before aggregation. This paper introduces In-network Generalized Trustworthy Data Collection (IGTDC) framework for event detection in CPS. This framework facilitates reliable data for aggregation at the edge of CPS. The main idea of IGTDC is to enable a sensor's module to examine locally whether the event's acquired data is trustworthy before transmitting towards the upstream nodes. It further validates whether the received data can be trusted or not before data aggregation at the sink node. Additionally, IGTDC helps to identify faulty sensors. For reliable event detection, we use collaborative IoT tactics, gate-level modeling with Verilog User Defined Primitive (UDP), and Programmable Logic Device (PLD) to ensure that the event's acquired data is reliable before transmitting towards the upstream nodes. We employ Gray code in gate-level modeling. It helps to ensure that the received data is reliable. Gray code also helps to distinguish a faulty sensor. Through simulation and extensive performance analysis, we demonstrate that the collected data in the IGTDC framework is reliable and can be used in the majority of CPS applications.

## Introduction

Due to the potentials of permeative surveillance, Internet of Things (IoT)-based sensing has appealed in different domains, such as industrial fabrication, supply chain, structural health monitoring, agriculture, fire detection, weather forecasting, and military observation (Liu et al., 2020a; Ali et al., 2020a). Currently, there are over 20 million devices connected to the IoT and Cyber-Physical Systems (CPS) (Liu et al., 2020a; Rahman et al., 2019a). By 2025, the worldwide economy is estimated to produce about USD 2.7 to 6.2 trillion per year contribution from the IoT (Manyika et al., 2013; Makhdoom et al., 2018).

Among all these aforementioned applications, one of the most significant application is sensor data collection, where sensors are used to monitor and collect various aspects of the surrounding environment and transfer the data towards upstream nodes for further processing (Alazab et al., 2020b; Tao et al., 2020; Zhang et al., 2020; Rahman et al., 2019a). However, sensors can be easily spoiled due to technical constraints and environmental impacts such as equipment failure, noises, absence of sensor calibration, and security concerns (Rahman et al., 2019a; Bhuiyan, Wang & Choo, 2016). Consequently, data collection in CPS/IoT is often unreliable. This makes detection of an event (e.g., structure health, fire detection) through data aggregation unreliable (Tao et al., 2020; Zafar et al., 2017; Bhuiyan, Wang & Choo, 2016). This dilemma is particularly acute in fire detection, structural health monitoring, and chemical explosions, where the system should be able to identify the collected data faults in real-time and take prompt recovery actions (Wang et al., 2020a, Zafar et al., 2017; Tao et al., 2020; Bhuiyan, Wang & Choo, 2016). For instance, an investigation reveals that approximately 93.4% of homes in the United Kingdom use fire detection systems (UK & HomeOffice, 2017). However, the analysis shows that there are still several losses, notwithstanding the fire detection systems. Furthermore, the study reveals that more than a dozen per million people still die each year. The study further proves that most of the accidents were caused by false alarms. Besides, most of the fires are not identified due to faulty sensors or sensors’ malfunction (Saeed et al., 2018).

Nevertheless, the reliability of event detection entirely depends on the data trustworthiness, such as if the data originates from a reliable source, is up-to-date, and accurate (Ali et al., 2021; Azzedin & Ghaleb, 2019; Zafar et al., 2017; Bertino, 2014). We employ different robust algorithms for data processing, transmitting, and storing, particularly when untrustworthy or unreliable is collected for various CPS events at the aggregating time. Consequently, decisions based on unreliable data may be meaningless, i.e., we may process, encrypt, transfer, and store unreliable data. Therefore, before investing time and resources on untrustworthy data, it is extremely desirable to collect, process, and transfer reliable data from a large size of unreliable data. Secondly, it is imperative to analyze the reliability of received data before doing aggregation at upstream nodes (e.g., sink/gateway) (Ali et al., 2021; Azzedin & Ghaleb, 2019; Zafar et al., 2017).

A moderate set of work proposes several methods and protocols related to reliable event detection and aggregation (Liu et al., 2020b; Azzedin & Ghaleb, 2019; Zafar et al., 2017; Alazab et al., 2014). Most existing data reliability tactics are commonly used in Wireless Sensor Networks (WSN). These strategies are less suitable for CPS environments, such as CPS circumstances being complex, heterogeneous, and highly dynamic (i.e., it includes cloud, fog, and IoT devices). Moreover, most existing event-based data collection and aggregation strategies (e.g., sum, weighted average, and voting) are primarily worked on data acquisition, processing, and aggregation, rather than data reliability (Tao et al., 2020). Therefore, the traditional schemes may not present real facts in terms of trustworthy or untrustworthy collected data.

This paper introduces In-network Generalized Trustworthy Data Collection (IGTDC) framework to solve the preceding problems. We provide the concept of trustworthy data collection in the CPS environment. For simplicity, as a representative application, we focus on two different CPS scenarios, namely Forest Fire Detection and Smart Irrigation System, using IGTDC Framework. Fire is a significant event of disasters that induce the destruction of valuable assets and lives (Saeed et al., 2018). It is estimated that around 4.8 billion animals died in Australia’s forest fires (Aguiar et al., 2012). Therefore, timely detection of fire is essential to evade significant disasters (Bailey et al., 2018). Similarly, the Smart Irrigation System is an IoT-based system capable of automating the irrigation process by analyzing soil moisture and climate conditions (like raining). It improves productivity and reduces water consumption (HydroPoint, 2020; Sisinni et al., 2018).

The main idea of IGTDC is to enable a sensors’ module to examine locally whether the acquired data is trustworthy (i.e., reliable) or not before forwarding towards upstream nodes. It further distinguishes whether the received data can be trusted or not before data aggregation at the sink. Besides, IGTDC helps to recognize a faulty or compromised sensor. For in-network and real-time reliable event detection, we use collaborative IoT tactics, gate-level modeling with Verilog User Defined Primitive (UDP), and Programmable Logic Device (PLD) (Ciletti, 1999), to make sure that the acquired data is reliable before transmitting towards upstream nodes. We employ Gray code (Abd El-Latif et al., 2018) in gate-level modeling. It helps resolve the uncertainty with multiple noisy sensors. Gray code also helps to distinguish a faulty sensor. As a result, IGTDC solely transmits reliable data towards upstream nodes and verify whether or not the received data is trustworthy before data aggregation. We envision that IGTDC gives in-network and low-cost solutions towards trustworthy data collection in the CPS environment. Furthermore, we emphasize faulty or compromised sensor detection to collect more reliable data and enhance system reliability. Moreover, based on the application requirements, IGTDC significantly reduces the amount of data uploaded to the cloud and helps overcome the network delay communication problem because a large amount of irrelevant data is filtered at the sensor’s module.

Our major contributions are as follows:

- We propose the IGTDC framework, which is a trustworthy data collection scheme in CPS. IGTDC can verify locally that the acquired data is trustworthy before transmitting towards upstream nodes and can verify that the received data is trustworthy before data aggregation.
- We present a tiny utility based on collaborative IoT technique and gate-level modeling that validate “acquired data” trustworthiness before transmitting towards upstream nodes.
- We propose to utilize the “Gray Code” tactic for data aggregation and faulty sensor detection. Gray Code can resolve the uncertainty with multiple sensors’ data before data aggregation and can trace a faulty or compromised sensor.
- We have performed an extensive performance analysis of IGTDC. The results prove the effectiveness of the IGTDC.

The rest of the paper is organized as follows. “Related Work” briefly discusses related work. “IGTDC: Trustworthy Data Collection Framework” briefly discusses the need for data trustworthiness in the CPS environment and emphasizes our proposed framework. “Acquire Data Trustworthiness Validation Before Transmission” illustrates trustworthy data collection approach. “Receive Data Trustworthiness Validation Before Aggregation” presents a trustworthiness validation approach before the data aggregation. “Performance Evaluation” evaluates our proposed framework. Lastly, we conclude and recommend future directions in “Conclusions”.

## Related Work

In the past decade, data trustworthiness is attracted by many domains, particularly healthcare, industrial fabrication, structural health monitoring, fire detection, and agriculture. Generally, a broad range of literature exists in the design of effective methods to ensure and improve data’s trustworthiness in various CPS applications (Kumar et al., 2021; Gupta et al., 2020; Alazab et al., 2020a; Ali et al., 2020b; Vasan et al., 2020; Tao et al., 2020). Based on the literature, articulate solutions need to be developed by combining different approaches and techniques to ensure data trustworthiness in sensors’ data life cycle (i.e., during data acquisition, transmission, aggregation, and cloud storage) (Hai et al., 2020; Gheisari et al., 2019; Rahman et al., 2019b; Zafar et al., 2017). However, data trustworthiness is challenging in the heterogeneous CPS environment, and there is no impeccable approach to the dilemma of data trustworthiness to ensure data reliability during the sensors’ data life cycle (Hai et al., 2020; Rahman et al., 2019b, Zafar et al., 2017). This paper is a preliminary endeavor at data trustworthiness in the context of forest fire detection and smart irrigation system.

Rahman et al. (2019b) reviewed all possible potential solutions for trustworthy data collections in the CPS environment. Furthermore, they highlighted all the challenges and proposed a data collection taxonomy for the CPS environment. Similarly (Bertino, 2014) also suggested an architectural framework for trustworthy data collection in the CPS environment. However, it does not describe how the framework can work in the heterogeneous CPS environment.

To address the data trustworthiness concerns, Li, Song & Zeng (2017) proposed a policy-based secure and trustworthy sensing system for IoT. In the proposed model, sensor data and IoT nodes’ reliability are assessed based on the recording history and the context using different policy rules. They identify the unreliable IoT devices by assessing their data reporting history. However, the proposed scheme suffers from high energy consumption and network bottleneck problems because a large amount of context information and redundant data are sent to the upstream nodes. Firoozi, Zadorozhny & Li (2018) suggested In-network data processing framework for processing sensors’ data in WSNs. They utilize a subjective logic-based trust approach for processing the sensed data. The proposed scheme can reduce or eliminate data redundancy problems and driving minimized resource utilization. However, it does not consider data trustworthiness during data acquisition and transmission.

Given the fact that the sensor data may be inherently noisy, Karthik & Anan (2017) suggested a sensor data scheme for assessing the sensors’ data using the temporal, spatial, and attribute data features. The suggested data scheme is hybrid, and it operates at the lower sensor node and the upstream sink node. The outcome reveals that the offered data scheme outperforms to reveal data trust and event detection efficiently. Nevertheless, this method does not consider the heterogeneity (i.e., multi-sensor environment) of data sources to find the data’s trustworthiness. To maintain data sources’ heterogeneity and data confidentiality when collecting, storing, and accessing IoT data, Wang, Xu & Yang (2018) presented a secure data managing technique for cloud-assisted IoT. They consider three types of trust, which can evaluate sensor and sink nodes’ behavior. However, this scheme does not consider the data’s reliability when it is collected or transmitted.

In order to disseminate sensor data from physical space in a timely and reliable way, Bhuiyan & Wu (2016) proposed a reliable and secure data collection model for event detection in WNS. The proposed scheme facilitates to provide trustworthy data for aggregation at sink of WNS. However, this model does not consider the heterogeneity of data sources.

Apart from these schemes, in the absence of ground truth, Blockchain technology has garnered much admiration in several domains due to its desirable properties such as it is immutable (i.e., data entered can never be changed/removed), auditable, scalable, and decentralized. Stranieri et al. (2021) investigated the impact of Blockchain technology in smart cities and agriculture. They critically reviewed some of the ongoing Blockchain-based projects in various CPS domains. They suggested Blockchain technology for specific objectives, such as assuring traceability or improving sales and reputation. Similarly, Ali et al. (2020) proposed a Blockchain-based framework for IoT data trustworthiness. However, they did not provide high level discussion about the used cases with no implementation details or results. Likewise, Ali et al. (2018) proposed a Blockchain-based model to ensure the trustworthiness of the wearable device registration and stored data in a cloud-assisted IoT. The proposed framework allows validating whether or not a device or sensor’s data has been altered.

Recently, Kumar et al. (2021) proposed a privacy-preserving model that consolidates blockchain and deep learning methods for smart agricultural drones. The proposed framework has several advantages. First, it is straightforward to use at the fogs and clouds and can efficiently distinguish most of the heterogeneous smart agriculture’s cyberattacks. However, Blockchain requires huge computing power, large memory, and high bandwidth due to control overheads in practice. Therefore, it is not suitable for deployment at the sensor level where computing, battery life, storage, and bandwidth are limited (Marchang, Ibbotson & Wheway, 2019). Still, Blockchain technology’s advantages are immense if Blockchain is applied in the upstream nodes such as fog/cloud level and considers data trustworthiness during data acquisition and before aggregation. Otherwise, untrustworthy or unreliable data can be collected at the fog/cloud level. Consequently, decisions based on unreliable data may be meaningless, i.e., we may process, transfer, and store unreliable data at the fog/cloud level.

In summary, most of the work similar to the above considers trusted computing and trust communication. However, data trustworthiness validation at acquisition and before/after transmissions are not considered. Moreover, most of the existing research focused on reducing the amount of data transmitted. Nevertheless, they did not consider in-network data processing and data reliability whether the acquired and transmitted data is reliable or not before aggregation, which is our research’s motivation. A preliminary step of the limitations as mentioned above is considered in the IGTDC framework. IGTDC can verify locally that the acquired data is trustworthy before transmitting towards upstream nodes (e.g., gateway) and verify that the received data is trustworthy before data aggregation.

## IGTDC: Trustworthy Data Collection Framework

This section briefly introduce the network and data models used. Then, we discuss our proposed IGTDC framework for trustworthy data collection in the CPS environment.

### Network architecture

**Figure 1:**
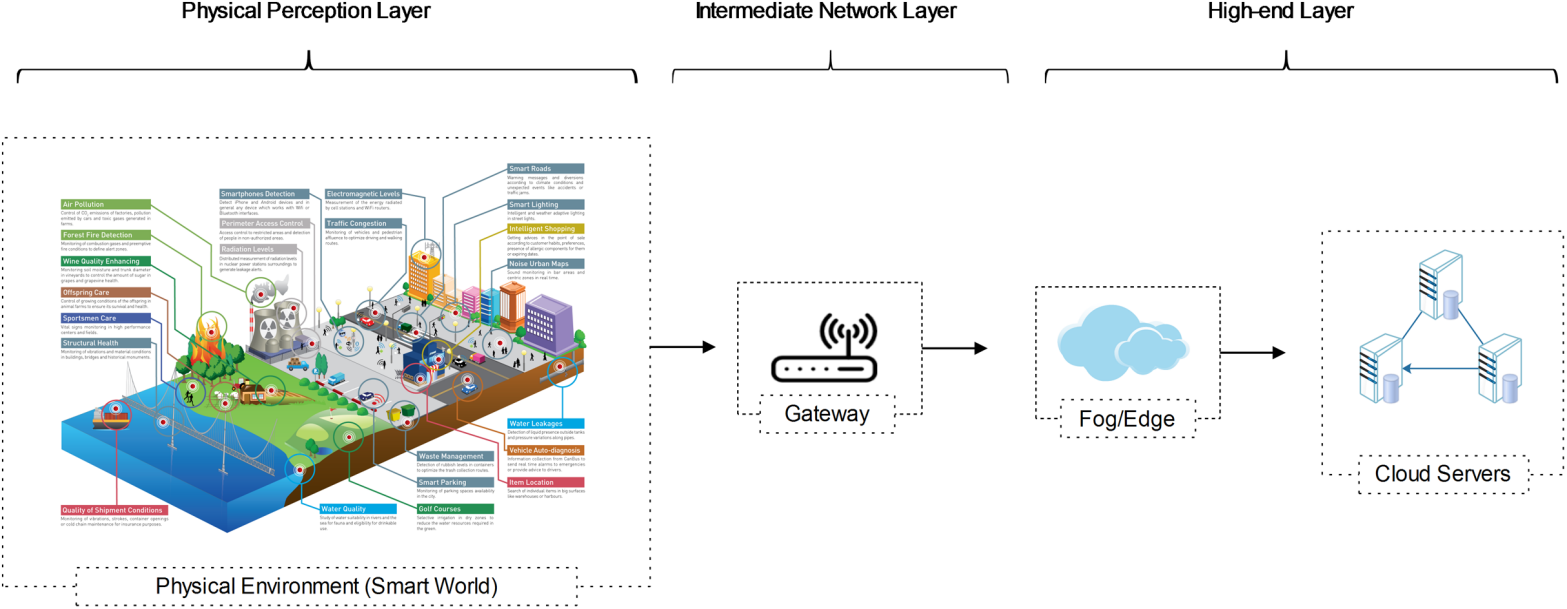
A generalized three-tier cloud-assisted CPS network architecture.

CPS and IoT are complementary models. Both utilize sensors to join all distributed intelligence to take a plentiful profound awareness of the environment and enabling more precise activities. Like traditional IoT networks, we consider a WSN with IoT and Cloud for CPS with data collecting, data transmitting, and high-level processing layers. We illustrate a representative three-tier Cloud-Assisted CPS network architecture in Fig. 1. The network architecture is designed like the traditional IoT data collection model:

- Physical perception layer (IoT devices level)
- IoT network layer (intermediate data processing level at cluster heads or aggregators)
- High-end layer (data processing and storage layer, such as fog and cloud)

As revealed in Fig. 1, the physical perception layer is used to perceive and estimate the surrounding environment. The interconnected sensors or mobile devices are used to monitor various features of the surrounding environment, e.g., movement, pressure, temperature, sleep habits, fitness levels, humidity, smoke, structure health, etc. (Zhang et al., 2018; Fremantle & Scott, 2017). The network layer processes the received data and transmits the collected data towards the upstreams nodes. The data can be further processed at the high-end layer and disclose to derive useful knowledge for various smart services (Sánchez-Gallegos et al., 2020; Gheisari, Wang & Chen, 2020; Wang et al., 2020b). To sum up, no matter how diverse the CPS application is, follow these five necessary steps: 1) Sense; 2) Transmit; 3) Store; 4) Analyze; 5) Act (Tao et al., 2020; Tianxing et al., 2020; Rahman et al., 2019a).

### Data model

As a representative application, we focus on two CPS scenarios, namely forest fire detection and smart irrigation system.

**Figure 2:**
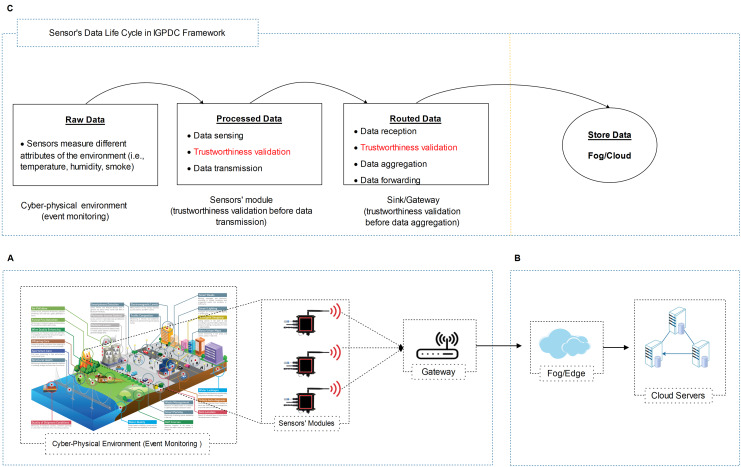
Generalized cloud-assisted CPS environment with IGTDC framework. (A) A trustworthy data collection scheme for event detection. (B) High-end data processing and storage layer (i.e., fog and cloud servers). (C) Sensor’s data life cycle in IGTDC framework. IGTDC can verify that the acquired data is trustworthy before transmitting towards upstream nodes (e.g., gateway) and verify that the received data is trustworthy before data aggregation.

In the case of forest fire detection, we reflect a hierarchical CPS environment with a set of sensors’ modules as shown in Fig. 2. In IGTDC, sensors’ modules are installed following engineering-driven deployment procedures. Sensors continuously monitor environmental quantities related to fire (e.g., temperature, smoke, and CO_2_) to evade significant disasters. Every sensor module collects the data from different sensors. If a sensor in the IGTDC needs to transmit any data, it forwards the collected data to the sensors’ module. The sensors’ module (before data transmission) verifies it and then sends the ultimate output (decision) towards the gateway. As a result, the collected data has been tested locally (in real-time) and forward towards the upstream nodes. Besides, the gateway provides facilities for different application platforms, verifies the received data, finally forwards the aggregated data to the fog/cloud.

Similarly, in smart irrigation, the same hierarchical CPS network architecture with a set of IGTDC sensors modules is used to continuously monitor soil conditions and the climate (i.e., temperature and humidity) to achieve better yields and save water. Through IGTDC, a simple Boolean expression can be modified according to smart irrigation specific requirements (also see “Data Reliability at Acquisition” for more details). That why we called this framework the “In-network and Generalized Trustworthy Data Collection (IGTDC)” framework because IGTDC is flexible, works at the edge of the network (e.g., sensors module level), and can be applied for the vast majority of CPS applications.

### Trustworthy data collection model

In traditional CPS/IoT environments, various sensors produce large amounts of data. A large volume of irrelevant, redundant, faulty, and noisy data are transmitted to the upstream nodes. Consequently, data fault, inaccuracy, and inconsistency can also occur (Tang, Alazab & Luo, 2017). Such unreliable data can lead to inaccurate analysis. Besides, direct data transmission to the upstream nodes is also not recommended due to latency, bandwidth, and device battery issues (Lv et al., 2020; Tao et al., 2020; Venkatraman & Alazab, 2018; Firoozi, Zadorozhny & Li, 2018).

Based on the CPS application, we assume many sensors’ modules are employed to collect trustworthy data. For trustworthy data collection, our foremost concerns are the CPS physical perception and intermediate network layers, as shown in Figs. 2A and 2C. As illustrated in Fig. 2C sensor’s data life cycle, a sensors’ module can verify locally that the acquired data is trustworthy before transmitting towards the upstream nodes. Furthermore, the sink or gateway can validate that the received data is trustworthy before data aggregation. As a result, reliable data is aggregated at the gateway. Secondly, a large amount of irrelevant data filtered at the sensors’ module level. Also, a reduced amount of data uploaded to the upstream nodes.

#### Data reliability at acquisition

Usually, data from sensors can be compromised at distinct stages, namely during the data acquisition, processing, transmission, aggregation, and storing. Among them, the first and most crucial is the acquisition stage. It is indispensable to ensure that the data acquired by the IGTDC is reliable. Moreover, we do not prefer to have compromised data at the time of acquisition due to data integrity, system integrity, sensor integrity, security attacks, and data manipulation problems.

**Figure 3:**
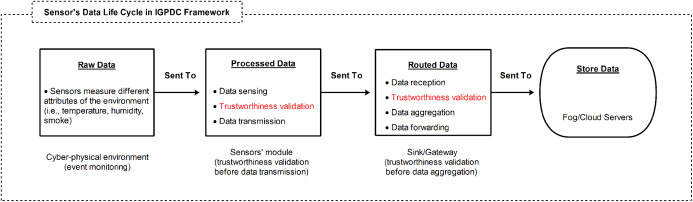
Sensor’s data life cycle and data trustworthiness validation in two levels (sensor’s module level and sink/gateway level) in the CPS: before/after data transmissions for event detection.

As shown in Fig. 3, for trustworthy data collection, we propose trustworthiness validation in two-level: at the sensor’s module-level (i.e., before data transmission) and sink/gateway-level (i.e., before data aggregation). We devise a tiny programmable logic device (PLD), which can operate at the edge level (i.e., CPS sensors’ module-level). We assume that the sensors’ module acquired data exploiting a state-space model. As a result, the acquired data has been tested locally (in real-time) without relying on trust reports from the upstream nodes (i.e., gateway or cloud) and forward reliable data to the upstream node such as a gateway.

Nevertheless, IGTDC’s PLD relies on digital combinational logic (i.e., Truth table, K-map, and gate-level implementations). This utility does not use memory owing to combinational and fewer logic elements (gate) (Mehta et al., 2016). Besides, PLD is based on simple Boolean expressions (i.e., Comparators, Sum of Product (SOP), and Product of Sum (POS)). A simple Boolean expression can be modified through PLD according to a specific CPS application, as Eqs. (1) and (4) are constructed and modified for the fire detection and smart irrigation system, respectively. Additionally, K-map (Mehta et al., 2016) is being used in the IGTDC logic design as an alternative representation of the truth table to assist in the minimal Boolean expression formulation. Moreover, K-map reduces logic functions faster and more efficiently than Boolean algebra (Rahman et al., 2019a, Mehta et al., 2016). By reducing, we intend to promote and reduce the number of logic gates and hardware inputs. We like to simplify the logic gates to the lowest cost to save costs by eliminating hardware components.

(1) $$Y_{1}=AC+AB+BC$$

(2) $$Y_{2}=AC+C(A⊕B)$$

(3) $$Y_{3}=A(B⊕C)+BC$$

(4) $$Y_{4}=CD+BD+BC+AD+AC+AB$$

For instance, Eqs. (1)–(3) can be used for the same event (fire detection) using different logic designs. K-map’s “don’t care” condition is a composite of inputs, and the designer does not concern about their output. Therefore, in Boolean expression formulation, “don’t care” conditions can either be involved or eliminated. Y1, Y2, and Y3 are three different minimal Boolean functions based on the logic domain truth table’s output. Where ‘A’, ‘B’, and ‘C’ stand for temperature, smoke, and CO_2_ sensors, respectively. The same concept can be applied for smart irrigation using Eq. (4), where ‘A’, ‘B’, ‘C’, and ‘D’ represent air temperature, air humidity, soil moisture, and soil temperature sensor, respectively. Equation (4), can also be further simplified, as we simplified Eq. (1) for fire detection. As a result, we envision that PLD gives a low cost and energy-efficient (due to reducing circuit switching and power consumption) and an in-network solution towards reliable data acquisition.

**Figure 4:**
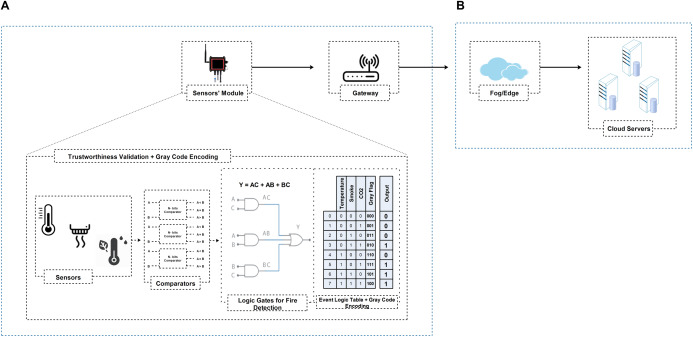
IGTDC framework for trustworthy fire detection. (A) Sensors’ module logic interpretation (i.e., comparators, logic gates, event logic table, and Gray flag information) for data trustworthiness validation in real-time without relying on trust reports from the upstream nodes (i.e., gateway or cloud). (B) High-end data processing and storage layer for additional data processing to derive useful knowledge for various smart services.

**Figure 5:**
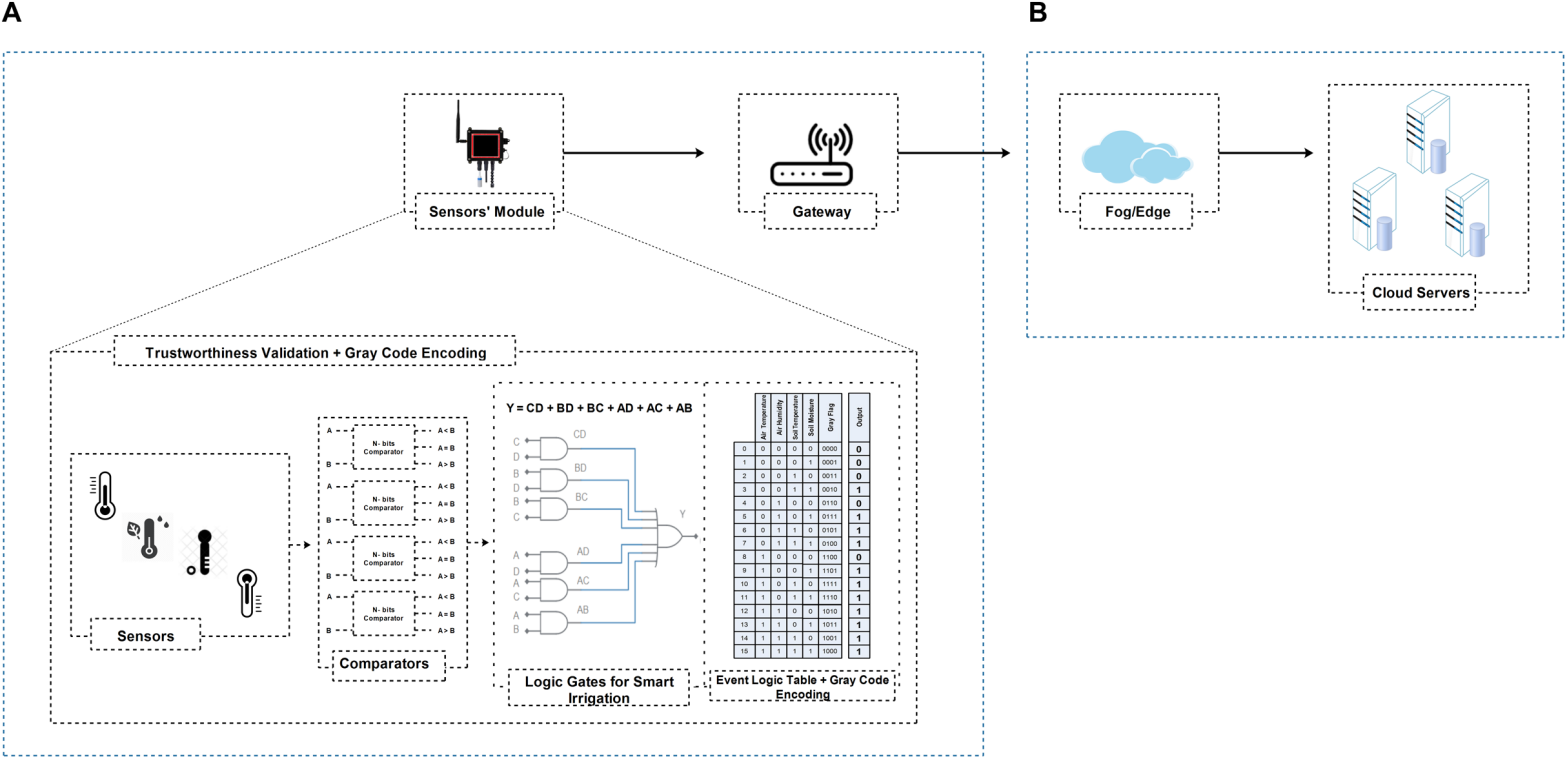
IGTDC framework for trustworthy smart irrigation system. (A) Sensors’ module logic interpretation (i.e., comparators, logic gates, event logic table, and Gray flag information) for data trustworthiness validation in real-time without relying on trust reports from the upstream nodes (i.e., gateway or cloud). (B) High-end data processing and storage layer for additional data processing to derive useful knowledge for various smart services.

In summary, according to Figs. 4A and 5A, PLD uses the logic domain inputs as primary roles for various operations and tests cases. The PLD module controls all the inside system actions and outputs. Besides, PLD outputs are like a record table, in which the Gray flag is used to records the history of all local decisions in a secret way during the monitor process (also read “Data Reliability at the Local Aggregator”, we have discussed Gray code in detail). As we have a total of 8 and 16 test cases for fire detection and intelligent irrigation, respectively, as shown in Figs. 4A and 5A event logic tables. According to the event logic tables, only reliable outputs are routed towards the gateway (represented by the ‘1’ output column) and the corresponding Gray flag. This trustworthy filtering and validation process can significantly reduce the amount of data upload to upstream nodes and help identify which sensor node participates in the event and vice versa.

#### Data reliability at the local aggregator

In our design, we utilize Gray code as secure data provenance and trust aggregation tactic, which can also be used to distinguish a faulty or compromised sensor.

As shown in Fig. 3 sensor’s data life cycle, after the data trustworthiness verification at the sensors’ module level, we often aggregate the collected data from a set of sensors at sink or gateway. The sensors’ data can be compromised after acquisition and during data transmission due to many factors such as: some sensors continually generate reliable data, while other sensors may provide biased, compromised, or fake data; compromised hardware sensors; sensors’ modules in the IGTDC may interfere with the sensors and the data may face integrity problem. Therefore, before data aggregation, second-level data trustworthiness validation can be applied by the local aggregator (i.e., gateway) to verify that the received data is trustworthy.

Below, we briefly discuss Gray Code as a data provenance and secure trust aggregation tactic. Gray code is defined as follows (Abd El-Latif et al., 2018):

(5) $$g_{i}=b_{i}⊕b_{i+1},$$

where *i* = 0,1,…,*n* − 1, and *b* = (*b*_*n*_*b*_*n*−1_…*b*_1_*b*_0_)

(6) $$g_{n}=b_{n}$$

Gray code is a ranking scheme for binary number systems in which two consecutive values differ in only one bit, which is also called Cyclic Code and Minimum Error Code. It is one of the standard signal coding practices in digital transformation. Moreover, it is commonly used in Boolean circuit minimization, high-speed decode circuits (i.e., to reduce circuit switching and save energy consumption), error correction, cryptography, and steganography (Abd El-Latif et al., 2018; Chen et al., 2015).

**Gray Code as data a provenance technique:** Data provenance plays a vital role in assuring data trustworthiness (Suhail et al., 2020). Since data are originated by multiple sensors’ modules and transmitted to the upstream nodes in IGTDC, by utilizing data provenance, we can trace the data source for data reliability validation, such as which sensor participated in the event detection and vice versa.

As discussed in “Data Reliability at the Local Aggregator”, PLD outputs are like a record table, in which the Gray flag is used to record the history of all local decisions secretly during the monitor process. In our scheme, in order to keep the data transmitted by the sensors’ module confidential, the Gray code sequence is integrated with the sensors modules’ output as a secure data provenance flag to overcome the suspicion or eavesdropping caused by cryptography, as shown in Figs. 4A and 5B “Event Logic Table”. It will securely help gateways, such as which sensors participate in event detection and vice versa. Besides, if an adversary eavesdrops on a particular sensor’s status, the adversary cannot directly track or disrupt that particular sensor. For instance, “100” in Fig. 4A “Event Logic Table”, secretly represents that all sensor participates in event detection. While the flag “111” secretly represents that one sensor does not participate in the event detection, which is the smoke sensor.

**Figure 6:**
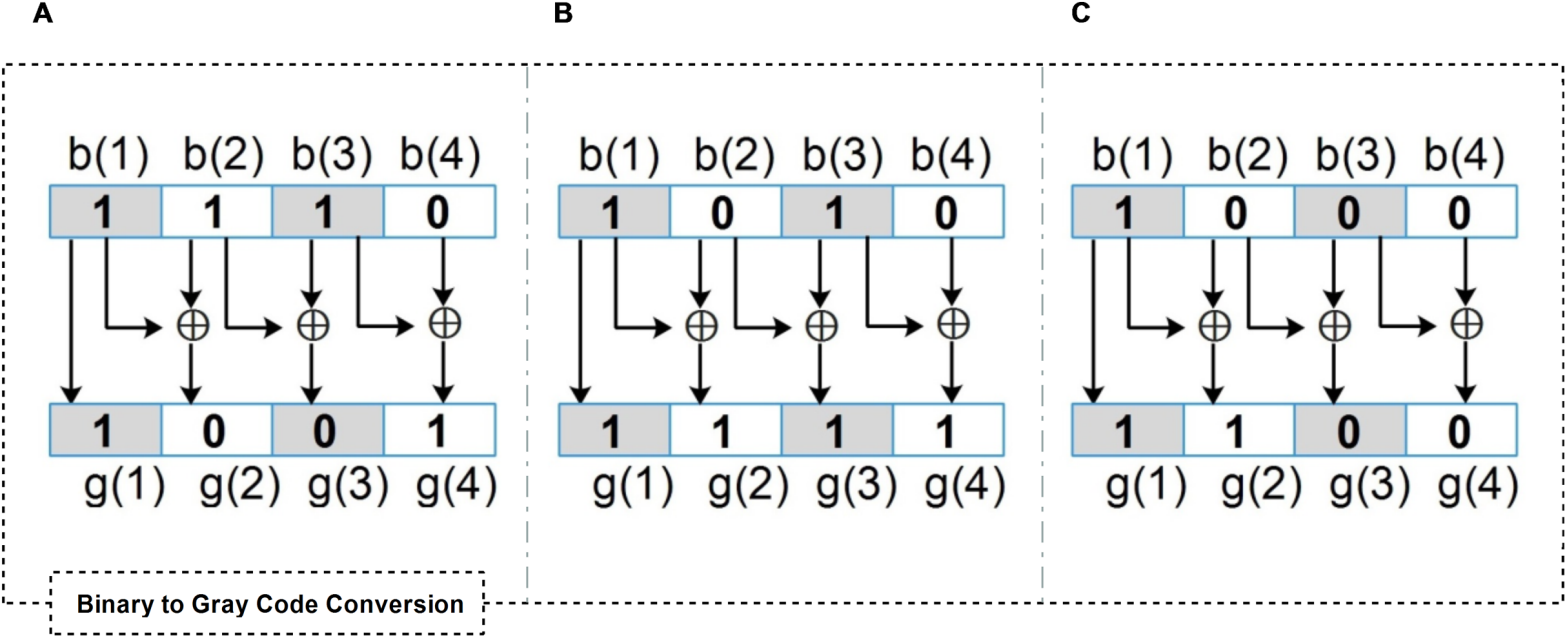
Different examples of binary to Gray code conversion over a 4-bits binary stream. (A) The flag “1001” represents that one sensor (i.e., b(4)) does not participate in the event detection. (B) The flag “1111” represents that b(2) and b(4) do not participate in the event detection (C) The flag “1100” represents that b(2), b(3), and b(4) do not participate in the event detection.

Figures 6A–6C show three different examples of binary to Gray code conversion over a 4-bits binary stream. Upon receiving a data packet integrated with the “Gray flag”, the gateway decodes the “Gray flag” and verifies the source and forwarding sensors’ module of an individual data packet since its generation.

In summary, a sensor’s module can make decisions locally without relying on trust reports from the upstream nodes (e.g., gateway or cloud), whether the acquired sensor’s data is trustworthy or not. If acquired data is reliable, it will be routed to the gateway. Upon receiving a packet at the gateway, it will validate the data packet trustworthiness before aggregation.

## Acquire Data Trustworthiness Validation Before Transmission

In this section, we discuss trustworthy data collection for reliable event detection. To make reliable event detection, we need trustworthy data collection in the CPS environment.

### Main idea

Generally, data from sensors can be compromised at different stages, namely during the data acquisition, processing, transmission, aggregation, and storing. Among them, the first and foremost stage is data acquisition. As mentioned earlier in “Data Reliability at Acquisition” the IGTDC’ PLD is adaptable and can be used in the majority of CPS applications. In this case, we consider two different CPS scenarios, namely fire detection and smart irrigation system using IGTDC framework.

In the case of a fire detection design prototype, sensors’ modules are deployed in three different areas to detect the right spot where the fire occurred. The sensors’ modules of the IGTDC framework are installed following an engineering-driven deployment technique to cover specific areas of interest. Fire-related incidents are monitored by three different sensors (e.g., temperature, smoke, and CO_2_). Afterward, using different comparators and PLD/UDP, the sensor’s raw data is compared with a predefined threshold, and ultimately, the corresponding reliable event data is routed to the upstream node ( i.e., gateway) for data aggregation as presented in Fig. 4.

A similar working principle of the IGTDC framework can be applied for other CPS applications. For instance, in the case of the smart irrigation system, as shown in Fig. 5, it almost similar to the fire detection system as shown Fig. 4. The difference lies in the number of sensors in two different situations (i.e., three and four different sensors for fire detection and smart irrigation system, respectively) and the corresponding comparators and logic gates.

### Identifying faulty/utrustworthy data

This subsection analyzes the acquired data for trustworthiness validation (i.e., whether or not the data is faulty or untrustworthy) before transmission to the upstream nodes.

To generate trustworthy event data/signal, we use a collaborative IoT strategy among sensors, gate-level modeling with Verilog User Defined Primitive (UDP), and Programmable Logic Device (PLD). As shown in Fig. 4, for fire detection, three different sensors acquire the temperature, smoke, and CO_2_ and forward them towards the PLD for local processing. Before PLD processing, all sensor data is considered raw data. Secondly, as shown in Fig. 4A, the sensors’ module utilizes different comparators and user-defined primitive (UDP) event logic table to process and evaluate all raw sensor readings. Each sensors module generate a reliable output according to Eq. (1), as shown in Fig. 4A “Logic Gates for Fire Detection”.

The output is considered reliable if and only if ‘n’ or (n−1) sensors detect an environmental event (i.e., ‘n’ or (n−1) sensors exceed a threshold at a given time), where ‘n’ represents the total number of sensors. Conversely, the output signal will be contemplated untrustworthy or compromised. For instance, as shown in Fig. 4A, we used three different fire detection sensors. If all sensors or at least two of the three sensors sense fire-related accidents in a specific time interval, the sensors’ module will route the trustworthy event information denoted by the ‘1’ output column, along with the corresponding Gray flag according to the event logic table.

Nevertheless, through the collaboration among sensors, a large amount of sensor data is filtered at the sensors’ module level, and only reliable data is routed towards upstream nodes. We refer to this data as the “routed data” processed by the sensors’ module, as shown in Fig. 3.

### Validation decision

The faulty or untrustworthy data verification can be performed in a distributed fashion where individual sensors’ module decides on the collected signals locally. The distributed approach simply needs the collaboration among sensor signals to be synchronized at each sensors’ module. Besides, the decision is almost fast and online since a sensors’ module does not rely on trust reports from the upstream nodes (e.g., gateway or fog/cloud).

The sensors’ module takes the final decision based on the information received from different sensors. After getting event information from two or more sensors, the sensors’ module generates a reliable output. If a single sensor’s reading contains an observed event, while at the same time other sensors did not detect the same event, it means observations from this sensor are more likely to be inaccurate or faulty. Using this scheme, the sensors’ module can know whether its collected data is trustworthy or not and solely transmits reliable event information towards upstream nodes.

## Receive Data Trustworthiness Validation Before Aggregation

In this section, we discuss received data or “routed data” trustworthiness validation before the data aggregation. Furthermore, we discuss how IGTDC recognizes a faulty or compromised sensor(s) using the Gray code provenance.

For trustworthy data aggregation, sensor data is validated at two different levels. The first data validation occurs on the sensors’ module (before data transmission) level, which has been discussed earlier in section. Once a sensors’ module has reliable data, it may be altered at the sensors’ module before/after transmission. This is to say, if the data is sent unprotected, the gateway is most likely to receive compromised (or altered) data for the aggregation. We should ensure that the acquired data originate from reputable sources and is not compromised during data transmission. Therefore, the second data trustworthiness validation occurs on the receiver/gateway node (before data aggregation). The second level trustworthiness validation scheme is due to the idea of data provenance, which can be used as evidence about the source of data, i.e., where and how the data is generated. In order to detect unreliable sensors or their unprotected data when receiving data, we use the Gray code provenance method in IGTDC, which helps to verify the authenticity of data based on the reliability of the data provider.

We perceive that traditionally distinct strategies have been suggested for resolving conflicts from multiple sources’ data. For instance, Data similarity, Mutual Information Independence (MII), Privacy-Preserving Data Mining (PPDM), Signal comparison, Signal correlation analysis, Truth discovery approach, Integrity models, and Quality of Context (QoC) are commonly used techniques for data aggregation as well as for resolving the conflicts with various noisy and cluttered sensors’ data (Azzedin & Ghaleb, 2019; Rahman et al., 2019b; Li et al., 2014; Huang et al., 2013). Nevertheless, in our approach, to check whether or not the received data is compromised or altered at the transmission, we used Gray code provenance technique. Notably, as discussed in “Data Reliability at the Local Aggregator”, it strengthens the gateway to examine unreliable sensors securely, such as which sensors participate in event detection and vice versa. If an adversary eavesdrops on a particular sensor’s status, the adversary cannot directly track or disrupt that particular sensor.

### Trustworthy data aggregation and provenance decoding

**Figure 7:**
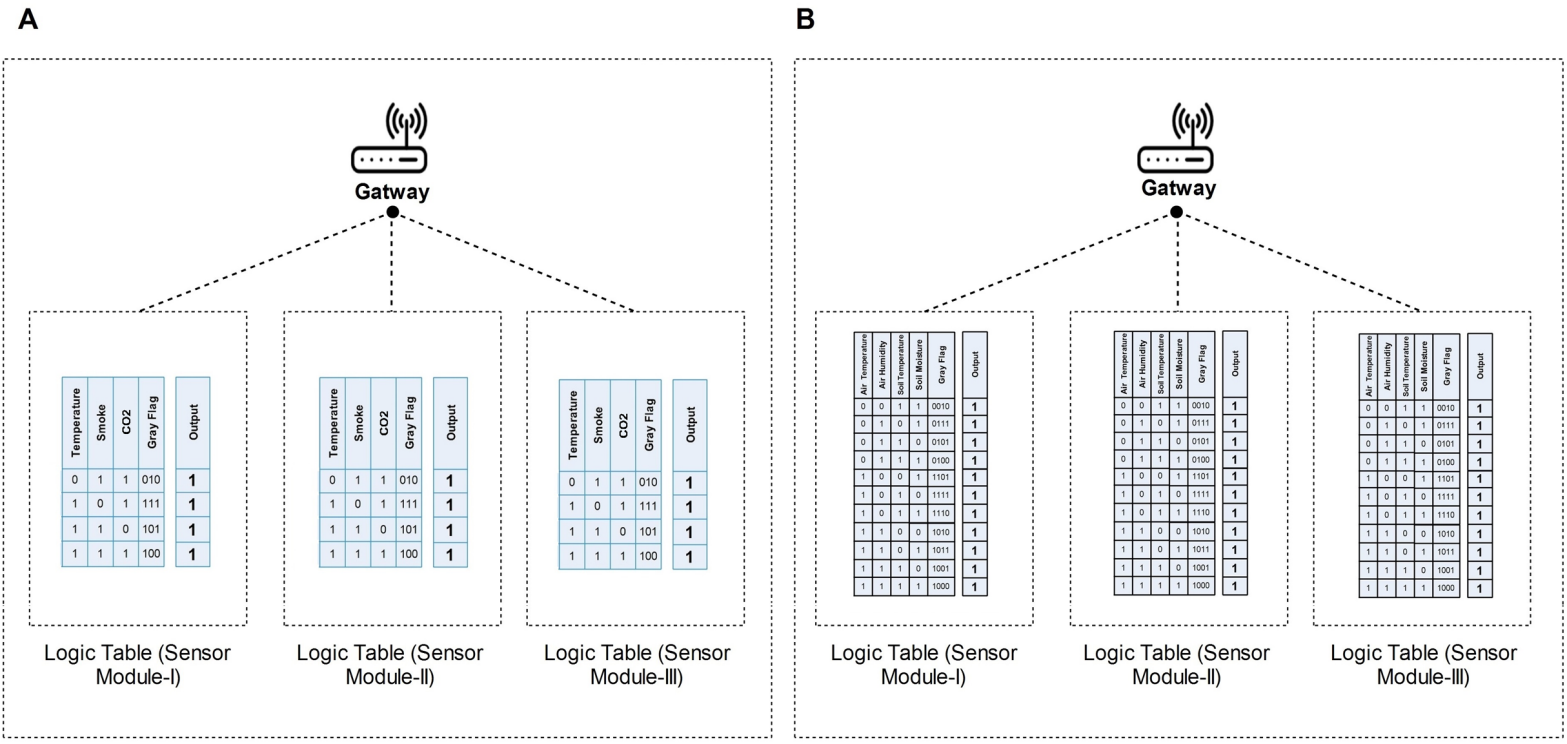
Figure 7 Sensors Modules’ reliable outputs and corresponding Gray flag at the Gateway. (A) shows all possible, reliable sensor outputs (i.e., four out of eight test cases) for three different sensors’ modules for fire detection. (B) shows all possible, reliable sensor outputs (i.e., 11 out of 16 test cases) for three different sensors’ modules for smart irrigation.

When the gateway receives a packet, it decodes the flag record for data validation before aggregation. In the case of fire detection, according to Fig. 4, the gateway receives reliable sensor outputs along with Gray flag sequence as shown in Fig. 7A logic tables. Figure 7A shows all possible, reliable sensor outputs (i.e., 4 out of 8 test cases) for three different sensors’ modules. Before aggregation, at first, the gateway decodes the source node provenance information as described in “Data Reliability at the Local Aggregator”. So, it learns that which sensors and module participate in particular event detection, and vice versa. For resolving conflicts from multiple sources, the gateway then simply takes the mean of each sensor’s data as the final decision. If any variation occurs in the routing data of all sensors’ modules, there is a probability that a sensor is defective or jeopardized. Using Gray flag provenance, the gateway can identify a compromised or faulty sensor.

Once a particular sensor becomes compromised, malfunctioned, or does not participate in monitoring, the gateway can directly identify it through Gray flag provenance. For instance, in the case of fire detection, all sensors in Module-I and Module-II observed an event and routed the data along with the Gray flag “100” to the gateway, “100” secretly represents that all sensor participates in event detection, as shown in Fig. 7A Module-I and Module-II logic tables. At the same time, the temperature sensor of Module-III does not participate in event detection. However, Module-III will route the output of two sensors and the corresponding Gray flag “010”, the code “010” secretly represents that one sensor does not participate in the event detection. The gateway decodes all the Gray flags that belong to that particular event (i.e.,100, 100, and 010) and determines which sensors participate in the event detection. As shown in the logic table of Fig. 7A Module-III “010”, the temperature sensor did not participate while others participate.

Similarly, in the case of smart irrigation, the same technique can be applied by the gateway. However, this time gateway will utilize the Fig. 7B logic tables for their data validation.

## Performance Evaluation

In this part, we evaluate the performance of IGTDC to demonstrate the effectiveness of trustworthy data collection through simulation.

### Simulation models

To check the performance evaluation and prove the proposed scheme’s validity, we have implemented our proposed scheme by utilizing Icarus Verilog (hardware description language) for PLD programming and FDS data set (McGrattan et al., 2013). The FDS is a Computational Fluid Dynamics (CFD) model developed by the National Institute of Standards and Technology (NIST) to simulate fires in different environments. We used a Core i7 system with 16 GB of RAM and 64 Bit Win 10 OS.

We simulated a multi-sensor environment using FDA with a sensing range of 350 × 250 × 100. The considered dataset collected data from 300 different sensors during the 60-minute simulation. Besides, we utilized the latest version of the Icarus Verilog (hardware description language) for PLD programming and used GTKWave to analyze digital sensors’ waveforms. GTKWave is a fully functional GTK+ based wave viewer for Unix and Win32 (Yadav, Rajak & Fathima, 2013; Han, Xu & Duan, 2009). Besides, it is used to study the results of different emulators and testing tools for debugging on a Verilog or VHDL simulation design.

The data generated by FDS during fire simulation is used in the IGTDC framework to check the efficiency of our work. We considered temperature, smoke, and CO_2_ sensor data in this simulation. For temperature, smoke, and CO_2_ sensors, the sensor’s initial inputs are 25 °C, 60 ppm, and 60 ppm, respectively.

We considered existing state-of-the-art truth-finding and voting techniques i.e., CRH (conflict resolution on heterogeneous data) (Li et al., 2014) and FTVM (fast and tolerant voting mechanism) (Huang et al., 2013) for performing a comparison. Both are used to reduce conflicts when making decisions based on received data. Usually, the assumption for voting systems is that all end devices are considered equally reliable and that the information associated with the highest number of occurrences is considered the correct answer. In contrast, truth discovery is the process of selecting an actual truth value for a data item when different data sources provide conflicting information on it.

### Performance measures

In order to evaluate the performance of IGTDC, we choose Data Reliability (in terms of detection), Absolute Error (AE), and Relative Error (ER) as performance metrics. The Data Reliability of detection is defined by the detection capability of the IGTDC, which can give us a reflection of how adequately the IGTDC can respond to compromised or alter data. AE is the difference between the estimated value and the actual ground true facts. By AE, we can find how much precisely the approach’s output differs from the ground true facts. ER is the ratio of AE to the actual trustworthy data, i.e., ground true measurement. Each simulation runs 35 times.

### Results

We have conducted three sets of simulations. In the initial set of simulations, we executed the proposed IGTDC scheme for trustworthy data acquisition (i.e., data reliability in terms of detection) and transmission. In order to make a sensor untrustworthy, we have randomly chosen a portion of the sensors and fed faulty signals into the sensor data acquisition model.

**Figure 8:**
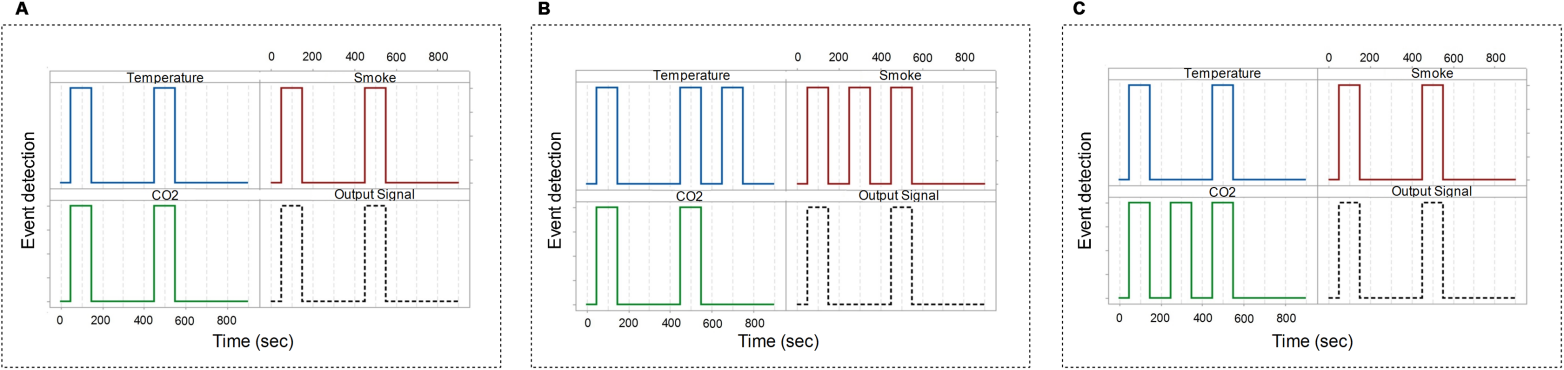
GTKWave waveform for trustworthy event detection using PLD/UDP. (A) GTKWave waveform for trustworthy (i.e., reliable) event detection without noise/faulty signals. (B) GTKWave waveform for trustworthy event detection with some noise/faulty signals. (C) GTKWave waveform for trustworthy event detection with some noise/faulty signals.

Figures 8A–8C show the digital sensors view waveform utilizing GTKWave, with diverse faulty sensor injection. Figure 8A shows no faulty or erroneous signal, which indicates that the acquired data is trustworthy (i.e., not modified by any signal failure or cyberattack), and the corresponding output (transmitted) signals are reliable. Figure 8B reveals that some sensors (i.e., temperature and a smoke sensor) having defective or somewhat compromised signals at 700 and 300 s, respectively. However, as shown in Fig. 8C, only one faulty signal is captured at the CO_2_ sensor. As a result, the faulty signal is discarded from the output signal, i.e., no output signal is generated for such a defective event. This confirms the accuracy of unreliable signal detection. We can see that there are different possible events. However, due to the collaboration of multiple sensors, only reliable events are detected. Whenever a sensors’ module gets such data, the sensors’ module drops the collected data before transmitting it to the upstream nodes. Because we do not take into account those outputs for which data is unavailable or compromise. As a result, it significantly reduces the amount of data uploaded to the upstream nodes. Further, it helps to overcome the network delay communication problem because a large amount of irrelevant data is filtered at the sensor’s module. We anticipate it will improve/save equipment battery, latency, and bandwidth problems.

**Figure 9:**
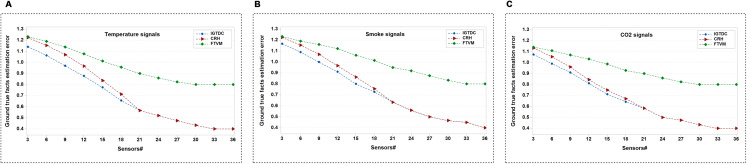
Data alternation and detection performance, under different random values of temperature, smoke, and CO_2_ for the IGTDC, CRH, and FTVM schemes with ground-fact estimates. (A) Ground true facts estimation using temperature signals. (B) Ground true facts estimation using smoke signals. (C) Ground true facts estimation using CO_2_ signals.

In the second set of simulations, we compare the correctness of ground true facts among IGTDC, CRH, and FTVM. Figures 9A–9C demonstrate the ground true facts approximation of IGTDC, CRH, and FTVM under different random values of temperature, smoke, and CO_2_, respectively. AE is utilized to measure the error. It can be seen that when the number of sensors varies from low to high, the performance of approximation error in IGTDC is better than that of CRH and FTVM. Furthermore, it is observed that the approximation error decreases as the number of sensors increases.

Additionally, the FTVM scheme shows inferior performance compared with IGTDC and CRH. One possible reason is that reliability estimation based on the maximum number of data packets or votes may not reveal real facts about fire detection systems. Such a Voting scheme’s unsatisfactory performance is that they assume that all packets from one sensor or all sensors are equally reliable. Consequently, the votes from diverse origins are uniformly weighted. Accordingly, this does not carry cognizance when the packets or votes are compromised or altered.

**Figure 10:**
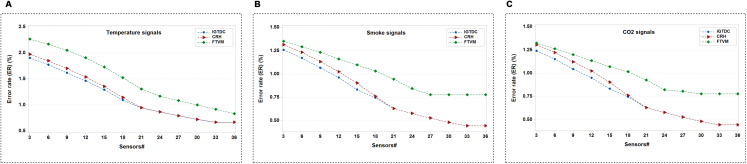
The number of sensors (i.e., temperature, smoke, and CO_2_) that sent trustworthy (i.e., reliable) data in different schemes. (A) Using temperature signals. (B) Using smoke signals. (C) Using CO_2_ signals.

In the final set of simulations, we consider ER in different schemes. We demonstrate the performance of IGTDC, CRH, and FTVM in terms of ER for three different sensors, as shown in Figs. 10A–10C. We can see that trustworthy data has been identified in all schemes. We can observe that the proposed scheme achieves a lower ER on the FDA data set than CRH and Voting. We can perceive that CRH scheme considers all the acquired data where some of the data are faulty or untrustworthy. In Voting, votes are used equally for all sensor nodes, and the sensor’s data is considered equally reliable. While IGTDC takes only reliable acquired and reliably received data. When a portion of the reliable data is altered at the acquisition or during transmission, this data is not included in the aggregation. As a result, the ER in IGTDC becomes lower than the other schemes.

Finally, the proposed IGTDC framework is compared with some other similar frameworks as shown in Table 1. In-network data processing is the first parameter to be considered. It refers to local validation of sensor data, i.e., real-time validation at the sensor module-level without trust reports from the upstream nodes. The next metrics are “trustworthiness validation before data transmission” and “trustworthiness validation before data aggregation”, they indicate whether the acquired data is reliable or not before forwarding towards upstream nodes and whether the received data can be trusted or not before data aggregation, respectively. The last two metrics are “multi-sensor environment” and “traceability”. The multi-sensor environment indicates whether the heterogeneity of sensor data is considered, while traceability ensure the scheme’s ability to track record history (i.e., the origin of the data record). The proposed design uses in-network data processing, two-level trustworthy data validation, and collaboration among sensors that ensure these properties. Besides, the proposed design has many advantages:

- It is easy to implement and deploy in the majority of CPS domains.
- The two-level trustworthy data validation significantly improved the overall performance of the proposed framework.
- In-network data processing and collaboration among sensors enable the proposed sensor modules to forward only reliable data and discard the defective or compromised data before transmitting it to upstream nodes. As a consequence, it significantly reduces the amount of data uploaded to the upstream node. Indirectly, this helps to overcome the network delay communication problem because a lot of irrelevant data is filtered in the sensor’s modules. We anticipate it to improve/save device battery, latency, and bandwidth issues.
- Due to provenance, the proposed scheme can identify a compromised sensor or faulty sensor’s data.

**Table 1:** Comparison between IGTDC and other state-of-the-art frameworks.

Authors	In-network data processing	Trustworthiness validation before data transmission	Trustworthiness validation before data aggregation	Multi-sensor environment	Traceability
Huang et al. (2013)	✗	✗	✓	✓	✓
Li et al. (2014)	✗	✗	✓	✓	✓
Bhuiyan & Wu (2016)	✗	✓	✓	✗	✗
Karthik & Anan (2017)	✓	✗	✓	✗	✗
Li, Song & Zeng (2017)	✗	✗	✓	✓	✗
Firoozi, Zadorozhny & Li (2018)	✗	✗	✗	✓	✗
Wang, Xu & Yang (2018)	✗	✗	✓	✓	✓
Ali et al. (2018)	✗	✗	✓	✓	✗
Ali et al. (2020)	✗	✗	✗	✓	✓
Kumar et al. (2021)	✗	✗	✓	✓	✓
Our approach	✓	✓	✓	✓	✓

## Conclusions

This paper has presented a trustworthy data collection framework for event detection in the CPS environment. The framework enables a sensors’ module to examine locally whether the acquired data is trustworthy before transmitting to upstream nodes. It further validates whether the received data can be trusted or not before data aggregation at the gateway. We used collaborative IoT strategies for trustworthy data collection and gate-level modeling using UDP/PLD to build a small utility program to ensure that the acquired data is reliable before transmitting towards upstream nodes. Besides, we utilize Gray code in gate-level modeling. It helps to ensure that the received data is reliable and can distinguish a faulty or compromised sensor. Finally, we have performed an extensive performance analysis of IGTDC. The results show that the proposed scheme’s data is reliable and can ensure reliable decision-making for event detection in the CPS environment. Our future direction is to provide data protection before data transmission towards the upstream nodes.

## Supplemental Information

10.7717/peerj-cs.504/supp-1 Supplemental Information 1 User manual to run the project code. This is the main document, in which we mention how to used and run the Icarus Verilog code.

10.7717/peerj-cs.504/supp-2 Supplemental Information 2 Example of comparator using UDP Logic Table; For event detection (Fire). This is Verilog File for Fire Detection.
Figure 9 (b) GTKWave waveform for Trustworthy Event Detection usingPLD/User Defined Primitive (UDP) (Logic Object Domain - I, Logic ObjectDomain - II, and Logic Object Domain- III) for Smart Irrigation System. Here we are using three different variables for three different sensors and two outputs one for the actual output for an event and one for the flag (i.e., Gray Code).
For more information such as how to run Verilog file and Testbench, kindly visit the below link for our project repository: https://github.com/hafizdir101/IGTDC-Project

10.7717/peerj-cs.504/supp-3 Supplemental Information 3 CaseOne TestBench for OurProjectFire.v module. This is the Testbench Verilog file and OurProjectFire.v module. We have 4 different Test Cases for fire detections such as shown in our main manuscript Figure 9 (a) GTKWave waveform for Trustworthy Event Detection using PLD/User Defined Primitive (UDP) (Logic Object Domain - I, Logic Object Domain - II, and Logic Object Domain- III) for fire detection and smart irrigation system.
For more information such as how to run Verilog file and Testbench, kindly visit the below link for our project repository: https://github.com/hafizdir101/IGTDC-Project

10.7717/peerj-cs.504/supp-4 Supplemental Information 4 CaseTwo testbench for OurProjectFire.v module. This is the Verilog testbench for OurProjectFire.v module.
Figure 10. (a) GTKWave waveform for Trustworthy Event Detection using PLD/User Defined Primitive (UDP) (Logic Object Domain - I) with some noise/faulty signals.
For more information such as how to run Verilog file and TestbenchCase2, kindly visit the below link for our project repository: https://github.com/hafizdir101/IGTDC-Project

10.7717/peerj-cs.504/supp-5 Supplemental Information 5 Case3 testbench for OurProjectFire.v module. This is a Verilog testbench for OurProjectFire.v module. Case 3 Testbench is used for Figure 10. (b) GTKWave waveform for Trustworthy Event Detection using PLD/User Defined Primitive (UDP) (Logic Object Domain - II) without noise/faulty signals.
For more information such as how to run Verilog files and Testbench, kindly visit the below link for our project repository: https://github.com/hafizdir101/IGTDC-Project

10.7717/peerj-cs.504/supp-6 Supplemental Information 6 Case4 Testbench for OurProjectFire.v module. This is Verilog testbench file for OurProjectFire Case 4.
Case 4: Figure 10. (c) GTKWave waveform for Trustworthy Event Detection using PLD/User Defined Primitive (UDP) (Logic Object Domain - III) with some noise/faulty signals.
For more information such as how to run Verilog file and Testbench, kindly visit the below link for our project repository: https://github.com/hafizdir101/IGTDC-Project

10.7717/peerj-cs.504/supp-7 Supplemental Information 7 Example of comparator using UDP Logic Table; For event detection (Smart Irrigation System). This is the Verilog module/code for Figure 9 (b) GTKWave waveform for Trustworthy Event Detection (Smart Irrigation System using PLD/User Defined Primitive (UDP) (Logic Object Domain - I, Logic Object Domain - II, and Logic Object Domain- III) for Smart Irrigation System.
For more information such as how to run Verilog file, kindly visit the below link for our project repository: https://github.com/hafizdir101/IGTDC-Project.

10.7717/peerj-cs.504/supp-8 Supplemental Information 8 Case 5: Varilog Testbench for OurProjectSAS.V module. This is a Verilog testbench file for trustworthy event detection (smart irrigation) for Figure 9 (b) GTKWave waveform for Trustworthy Event Detection usingPLD/User Defined Primitive (UDP) (Logic Object Domain - I, Logic Object Domain - II, and Logic Object Domain- III) for Smart Irrigation System.
In this simulation, we can observe three out of four reliable events are detected at 39,600, 81,200, and 16,400 s, respectively, over 173,200 s (48.1 Hours) of simulation time.
For more information such as how to run Verilog file and Testbench, kindly visit the below link for our project repository: https://github.com/hafizdir101/IGTDC-Project.

## Additional Information and Declarations

### Data Availability

The following information was supplied regarding data availability: Data is available at GitHub (IGTDC-Project): https://github.com/hafizdir101/IGTDC-Project.
