# Supplementary material for: In-network generalized trustworthy data collection for event detection in cyber-physical systems
Source: PeerJ Comput Sci. 2021 May 4;7:e504. doi: 10.7717/peerj-cs.504 (PMC8114821; doi:10.7717/peerj-cs.504)
Supplement: Supplemental Information 1 — This is the main document, in which we mention how to used and run the Icarus Verilog code. [file peerj-cs-07-504-s001.pdf]

Icarus Verilog code implementation for Trustworthy for Event Detection (Fire detection and Smart Irrigation System).

This document is based upon free Icarus Verilog compiler, that works very well for windows as well as Linux. We use Intel Core i7, 16 GB RAM, Win 10, 64 Bit OS. We utilised the latest version of the Icarus Verilog (hardware description language version 10.3) for PLD programming, and GTKWave (version 3.3.104). This is a very small footprint software, especially if we wish to eventually port the code in a real FPGA and see the things working in real - and not just in simulator.

In order to run our project code, first we need to know about the Icarus Verilog fundamental.

Introduction

Hardware Description Language (HDL) is used to model digital circuits using codes. Verilog is one such code (VHDL is another type). Icarus Verilog is a free compiler implementation for the IEEE-1364 Verilog hardware description language. Icarus is maintained by Stephen Williams and it is released under the GNU GPL license.

What Is Icarus Verilog?

Icarus Verilog is a Verilog simulation and synthesis tool. It operates as a compiler, compiling source code written in Verilog (IEEE-1364) into some target format. For batch simulation, the compiler can generate an intermediate form called vvp assembly. This intermediate form is executed by the ``vvp'' command. For synthesis, the compiler generates netlists in the desired format. The compiler proper is intended to parse and elaborate design descriptions written to the IEEE standard IEEE Std 1364-2005. This is a fairly large and complex standard, so it will take some time to fill all the dark alleys of the standard, but that's the goal.

A Test Suite?

There is also a test suite available. The test suite is also accessible as the ivtest github.com project, available here:

<https://github.com/steveicarus/ivtest>.

Icarus Verilog for Windows

You can find Icarus Verilog sources and binaries for most platforms at the Icarus site FTP. The sources available here:

http://bleyer.org/icarus/

How to run your first Verilog code/program?

Below is a complete tutorial:

Link: http://www.referencedesigner.com/tutorials/verilog/verilog_01.php

The C:/iverilog/bin subdirectory contains the executable file verilog.exe that is used to run simulator.

Now create a new file called hello.v in the directory C:/iverilog/bin and edit it with notepad or any other text editor(I prefer free notepad++). Enter the following lines of code in hello.v

```
module main;
  initial
    begin
      $display("Learning Verilog is easy with referencedesigner.com tutorial");
      $finish ;
    end
endmodule
```

- Go to your DOS prompt (Start - > cmd) and navigate to the directory C:/iverilog/bin

```
C:\> cd \iverilog\bin
```

Run iverilog using the command

```
C:\>iverilog\bin > iverilog hello.v
```

If you have done everything right, it should generate a file called a.out which you can run using command

```
C:\>iverilog\bin > vvp a.out
```

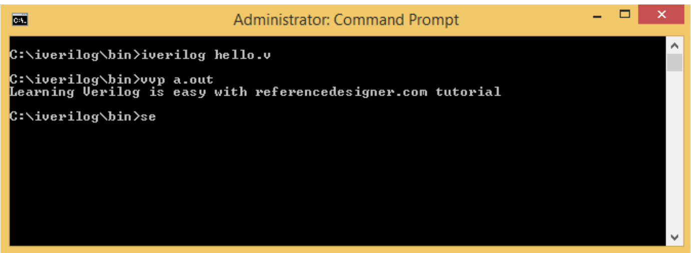

Every Verilog implementation goes through extensive verification. At this point the code just gives out the output saying "Learning Verilog is easy with referencedesigner.com tutorial". As shown in the above picture. It just verifies that your set up is ready for running Verilog code. Now we have understood the concept we will compile the code and run it in Icarus. In the next page we will write an actual real-life Verilog code two different IoT scenarios, i.e., for fire detection and smart irrigation system. Copy the following source codes in the directory C:\iverilog\bin.

****

Our Project Code and modules:

myproject.v

```
// Example of comparator using UDP Table; For event detection (Fire).
Case 5: Figure 9 (b) GTKWave waveform for Trustworthy Event Detection using
PLD/User Defined Primitive (UDP) (Logic Object Domain - I, Logic Object
Domain - II, and Logic Object Domain- III) for Smart Irrigation System.
//Here we are using three different variables for three different sensors
and two outputs one for the actual output for an event and one for the flag
(i.e., Gray Code). We use Intel Core i7, 16 GB RAM, Win 10, 64 Bit //OS. We
utilised the latest version of the Icarus Verilog iverilog-0.9.7 // (latest
stable release) for PLD programming, and GTKWave (version 3.3.104).
```

```
`timescale 1ns / 1ps
module comparator(
      input Temperature,
      input Smoke,
      input Humidity,
      output Output,
      output [2:0] Flag
);
compare c0(Output, Temperature, Smoke, Humidity);
      compare2 c20(Flag, Temperature, Smoke, Humidity);
      endmodule
      ///The below logic table is used for the logic gate design
      primitive compare(Output, in1, in2, in3);
            output Output;
            input in1,in2,in3;
      table
      // in1  in2  in3 : out
          0    0    0  :  0;
          0    0    1  :  0;
          0    1    0  :  0;
```

```
          0    1    1  :  1;
          1    0    0  :  0;
          1    0    1  :  1;
          1    1    0  :  1;
          1    1    1  :  1;

      endtable
      endprimitive
      // The below logic table is used for the Flag (i.e., Gray Code), it
//also transfer the status of all sensors such as which sensor or variables
//participate in the output
      primitive compare2(Flag, in1, in2, in3);
            output Flag;
            input in1,in2,in3;
      table
      // in1  in2  in3 : out2
          0    0    0  :  0;
          0    0    1  :  1;
          0    1    0  :  1;
          0    1    1  :  1;
          1    0    0  :  1;
          1    0    1  :  1;
          1    1    0  :  1;
          1    1    1  :  1;

      endtable
      endprimitive
```

CaseOne_tb.v

Note: CaseOne_tb.v is a testbench for myproject.v module; we have 5 different Test Cases for fire detections and smart irrigation such as shown in our main manuscript Figure 9 GTKWave waveform for Trustworthy Event Detection using PLD/User Defined Primitive (UDP) (Logic Object Domain - I, Logic Object Domain - II, and Logic Object Domain- III) for fire detection and smart irrigation system.

Case 1: Figure 9 (a) GTKWave waveform for Trustworthy Event Detection using PLD/User Defined Primitive (UDP) (Logic Object Domain - I, Logic Object Domain - II, and Logic Object Domain- III) for fire detection.

```
//CaseOne TestBench for myproject.v module
The simulation runs for 250 seconds According to the logical object module
for fre detection, four of the eight output values are sent towards the
gateway for the reliable event detection, denoted in the logic table by the
'1' output column. The reliable event information will be sent towards the
```

upstream node, if and only if, at least two sensors or all sensors sense an event simultaneously, i.e., temperature, smoke, and humidity sensor exceed the threshold. For trustworthy fire detection, we can observe two of the three reliable events are detected at 60 and 180 seconds, respectively, over 250 seconds of simulation time.

```
// testbench for myproject.v module
`timescale 1s / 1ps
module stimulus;
      // Inputs
      reg Temperature;
      reg Smoke;
      reg Humidity;
      // Outputs
      wire Output;
            wire [2:0] Flag;
            // Instantiate the Unit Under Test (UUT)
            comparator uut (
                  .Temperature(Temperature),
                  .Smoke(Smoke),
                  .Humidity(Humidity),
                  .Output(Output),
                  .Flag(Flag)
            );
            initial begin
                  // Initialize Inputs
            $dumpfile("test.vcd");
          $dumpvars(0,stimulus);
            Temperature = 0;
            Smoke = 0;
            Humidity = 0;
          // an event occures and all sensors observed the event
            #60 Temperature = 1;
            #0  Smoke = 1;
            #0  Humidity = 1;
            #20 Temperature = 0;
            #0  Smoke = 0;
            #0  Humidity = 0;
            ////////////only one sensor due to change environment effects
            #40 Temperature = 1;
            #0  Smoke = 0;
            #0  Humidity = 0;
            #20 Temperature = 0;
            #0  Smoke = 0;
            #0  Humidity = 0;
            ////////////only one sensor changes due to environment effects
            #10 Temperature = 0;
            #0   Smoke = 0;
            #0   Humidity = 1;
```

```
            #20 Temperature = 0;
            #0   Smoke = 0;
            #0   Humidity = 0;
            // an event occurs and all sensors observed the event
            #10 Temperature = 1;
            #0   Smoke = 1;
            #0   Humidity = 1;
            #20 Temperature = 0;
            #0   Smoke = 0;
            #0   Humidity = 0;
            #50;
          end
            initial begin
            $monitor("Time=%3d, Temperature=%d,  Smoke=%d,  Humidity=%d, Output=%d,  Flag (8421)=%b\n",$time, Temperature, Smoke, Humidity, Output, Flag);
            end
      Endmodule
      // C:\iverilog\bin> iverilog -o CaseOne myproject.v CaseOne_tb.v
      //C:\iverilog\bin>vvp CaseOne
      //C:\iverilog\bin>gtkwave test.vcd &
```

Now go to the dos windows (Start -> cmd) navigate to the iverilog\bin directory

C:\> cd iverilog\bin

Compile the program using

C:\iverilog\bin>iverilog -o CaseOne myproject.v CaseOne_tb.v

If everything goes right, it will not produce any output. If there are any syntax errors it will show some errors.

To see the output of the stimulus, you may like to give the following command

C:\iverilog\bin>vvp CaseOne

Note: If you want to see the output in the graphic form i.e., GTKWave then used below command

C:\iverilog\bin>gtkwave test.vcd &

This shows the following outputs.

Note: The above commands can be used for Case 2, Case 3, and Case 4 according to TestBench names.

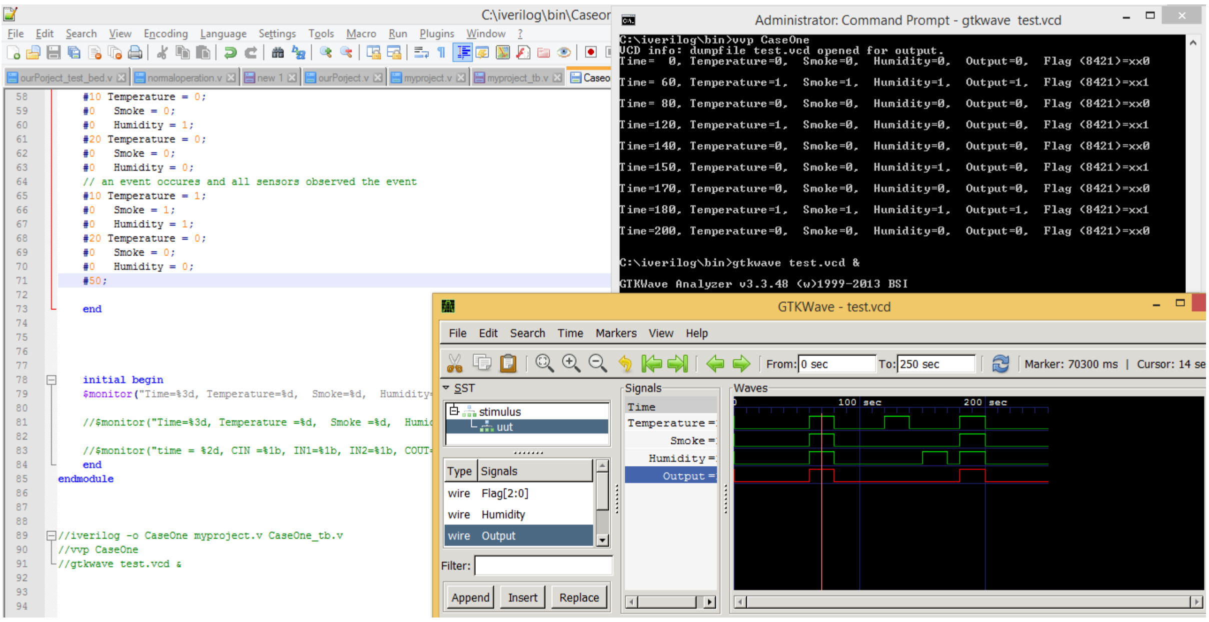

CaseTwo_tb.v

Case 2: Figure 10. (a) GTKWave waveform for Trustworthy Event Detection using PLD/User Defined Primitive (UDP) (Logic Object Domain - I) with some noise/faulty signals.

//The simulation runs for 870 seconds According to the logical object module for fire detection, four of the eight output values are sent towards the gateway for the reliable event detection, denoted in the logic table by the '1' output column. An output event is produced if and only if at least two of the three or all sensors observe the event simultaneously. For trustworthy fire detection, we can observe two out of four reliable events are detected at 150 and 500 seconds, respectively, over 870 seconds of simulation time. Furthermore, in this simulation the temperature and a smoke sensor having defective or somewhat compromise signals at 700 and 300 seconds, respectively.

```

// testbench for myproject.v module
`timescale 1s / 1ps
module stimulus;
        // Inputs
        reg Temperature;
        reg Smoke;
        reg Humidity;
        // Outputs
                wire Output;
                wire [2:0] Flag;
                // Instantiate the Unit Under Test (UUT)
                comparator uut (
                        .Temperature(Temperature),
                        .Smoke(Smoke),
```

```
                        .Humidity(Humidity),
                        .Output(Output),
                        .Flag(Flag)
                );
                initial begin
                        // Initialize Inputs
                $dumpfile("test.vcd");
            $dumpvars(0,stimulus);
                Temperature = 0;
                Smoke = 0;
                Humidity = 0;
                // an event occures and all sensors observed the event
                #150 Temperature = 1;
                #0  Smoke = 1;
                #0  Humidity = 1;
                #20 Temperature = 0;
                #0  Smoke = 0;
                #0  Humidity = 0;
                ////////////only one sensor change due to enviroment effects
                #0 Temperature = 0;
                #130  Smoke = 1;
                #0  Humidity = 0;
                #0 Temperature = 0;
                #20  Smoke = 0;
                #0  Humidity = 0;
           // an event occures and all sensors observed the event
                #180 Temperature = 1;
                #0   Smoke = 1;
                #0   Humidity = 1;
                #20 Temperature = 0;
                #0   Smoke = 0;
                #0   Humidity = 0;
                ////////////only one sensor change due to enviroment effects
                #180 Temperature = 1;
                #0   Smoke = 0;
                #0   Humidity = 0;
                #20 Temperature = 0;
                #0   Smoke = 0;
                #0   Humidity = 0;
                #150;
            end

                initial begin
                $monitor("Time=%3d, Temperature=%d,  Smoke=%d,  Humidity=%d, Output=%d,  Flag (8421)=%b\n",$time, Temperature, Smoke, Humidity, Output, Flag);
        Endmodule
```

```
        // C:\iverilog\bin> iverilog -o CaseTwo myproject.v CaseTwo_tb.v
        //C:\iverilog\bin>vvp CaseTwo
        //C:\iverilog\bin>gtkwave test.vcd &
```

Output:

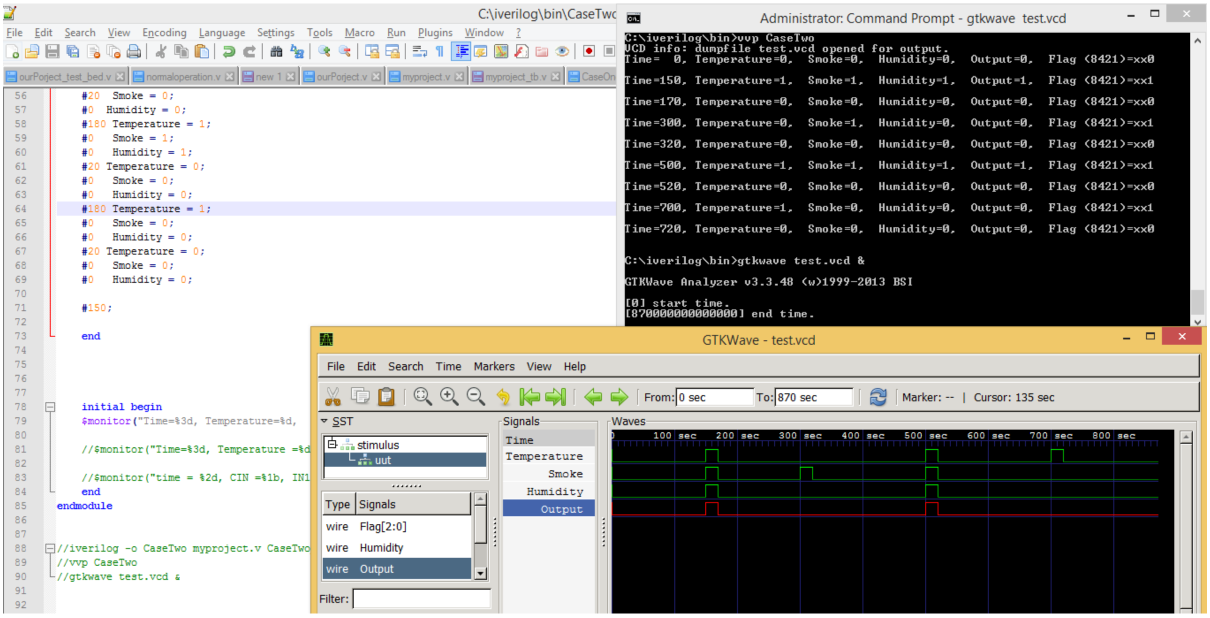

CaseThree_tb.v

Case 3: Figure 10. (b) GTKWave waveform for Trustworthy Event Detection using PLD/User Defned Primitive (UDP) (Logic Object Domain - II) without noise/faulty signals.

```
// CaseThree_tb.v Case 3: Figure 10. (b)
For trustworthy fire detection, we can observetwo out of four reliable events
are detected at 150 and 500 seconds, respectively, over 870 seconds of
simulation time. Furthermore, in this simulation we have no defective or
somewhat compromise signals, it means that the obtained event is trustworthy,
and the corresponding output signals are reliable.
```

```
// testbench for myproject.v module
`timescale 1s / 1ps
module stimulus;
        // Inputs
        reg Temperature;
        reg Smoke;
        reg Humidity;
        // Outputs
        wire Output;
                wire [2:0] Flag;
                // Instantiate the Unit Under Test (UUT)
                comparator uut (
                        .Temperature(Temperature),
```

```
                        .Smoke(Smoke),
                        .Humidity(Humidity),
                        .Output(Output),
                        .Flag(Flag)
                );
                initial begin
                        // Initialize Inputs
                $dumpfile("test.vcd");
            $dumpvars(0,stimulus);
                Temperature = 0;
                Smoke = 0;
                Humidity = 0;
                //an event occures and all sensors observed the event
                #150 Temperature = 1;
                #0  Smoke = 1;
                #0  Humidity = 1;
                #20 Temperature = 0;
                #0  Smoke = 0;
                #0  Humidity = 0;
                #0 Temperature = 0;
                #130  Smoke = 0;
                #0  Humidity = 0;
                #0 Temperature = 0;
                #20  Smoke = 0;
                #0  Humidity = 0;
                //an event occures and all sensors observed the event
                #180 Temperature = 1;
                #0   Smoke = 1;
                #0   Humidity = 1;
                #20 Temperature = 0;
                #0   Smoke = 0;
                #0   Humidity = 0;
                #180 Temperature = 0;
                #0   Smoke = 0;
                #0   Humidity = 0;
                #20 Temperature = 0;
                #0   Smoke = 0;
                #0   Humidity = 0;

                #150;

            end
                initial begin
                $monitor("Time=%3d, Temperature=%d,  Smoke=%d,  Humidity=%d, Output=%d,  Flag (8421)=%b\n",$time, Temperature, Smoke, Humidity, Output, Flag);
                end
        endmodule
```

```
// C:\iverilog\bin> iverilog -o CaseThree myproject.v CaseThree_tb.v
// C:\iverilog\bin> vvp CaseThree
// C:\iverilog\bin> gtkwave test.vcd &
```

Output:

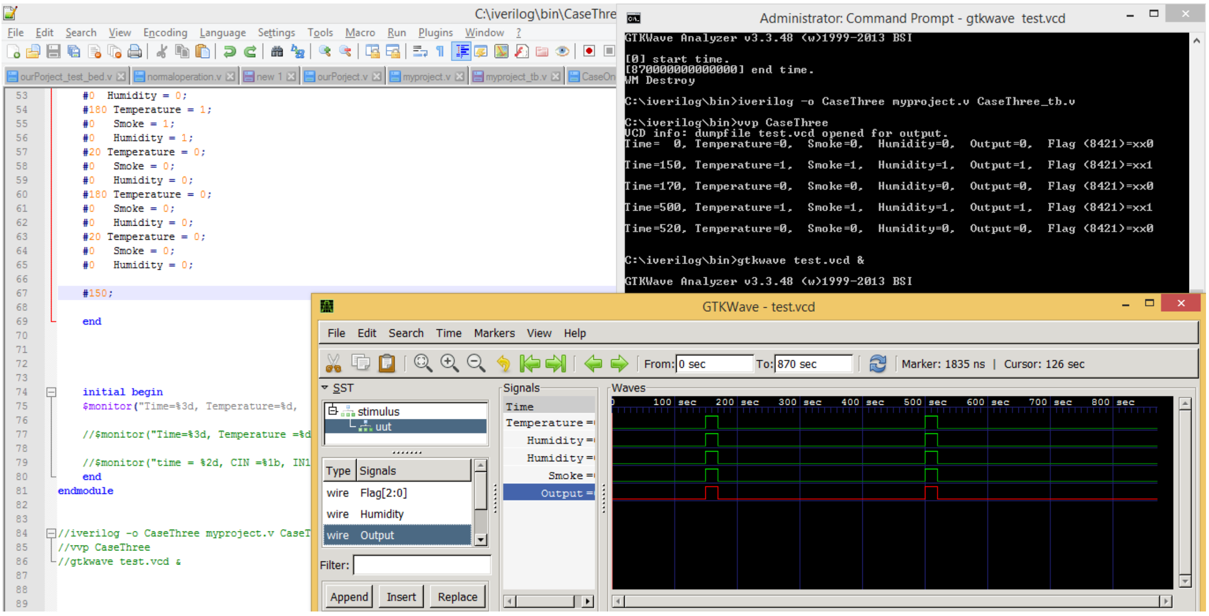

CaseFour_tb.v

Case 4: Figure 10. (c) GTKWave waveform for Trustworthy Event Detection using PLD/User Defned Primitive (UDP) (Logic Object Domain - III) with some noise/faulty signals.

```
// CaseFour_tb.v; For trustworthy fre detection, we can observe
// two out of four reliable events are detected at 150 and 500 seconds, respectively, over 870 seconds of simulation time. In this simulation, only one faulty signal is fed into the humidity sensor, and the associated event information is captured.
```

```
// testbench for myproject.v module
`timescale 1s / 1ps
module stimulus;
        // Inputs
        reg Temperature;
        reg Smoke;
        reg Humidity;
        // Outputs
        wire Output;
                wire [2:0] Flag;
```

```
        // Instantiate the Unit Under Test (UUT)
        comparator uut (
                .Temperature(Temperature),
                .Smoke(Smoke),
                .Humidity(Humidity),
                .Output(Output),
                .Flag(Flag)
        );

        initial begin
                // Initialize Inputs
        $dumpfile("test.vcd");
    $dumpvars(0,stimulus);
        Temperature = 0;
        Smoke = 0;
        Humidity = 0;
        //an event occures and all sensors observed the event
        #150 Temperature = 1;
        #0  Smoke = 1;
        #0  Humidity = 1;
        #20 Temperature = 0;
        #0  Smoke = 0;
        #0  Humidity = 0;
        ////////////only one sensor change due to enviroment effects
        #0 Temperature = 0;
        #0  Smoke = 0;
        #130  Humidity = 1;
        #0 Temperature = 0;
        #0  Smoke = 0;
        #20 Humidity = 0;
        //an event occures and all sensors observed the event
        #180 Temperature = 1;
        #0   Smoke = 1;
        #0   Humidity = 1;
        #20 Temperature = 0;
        #0   Smoke = 0;
        #0   Humidity = 0;
        #180 Temperature = 0;
        #0   Smoke = 0;
        #0   Humidity = 0;
        #20 Temperature = 0;
        #0   Smoke = 0;
        #0   Humidity = 0;

        #150;

    end

```

```
            initial begin
            $monitor("Time=%3d, Temperature=%d,  Smoke=%d,  Humidity=%d, Output=%d,  Flag (8421)=%b\n",$time, Temperature, Smoke, Humidity, Output, Flag);

            end
     endmodule

     // C:\iverilog\bin> iverilog -o CaseFour myproject.v CaseFour_tb.v
     // C:\iverilog\bin> vvp CaseFour
     // C:\iverilog\bin> gtkwave test.vcd &
```

Output:

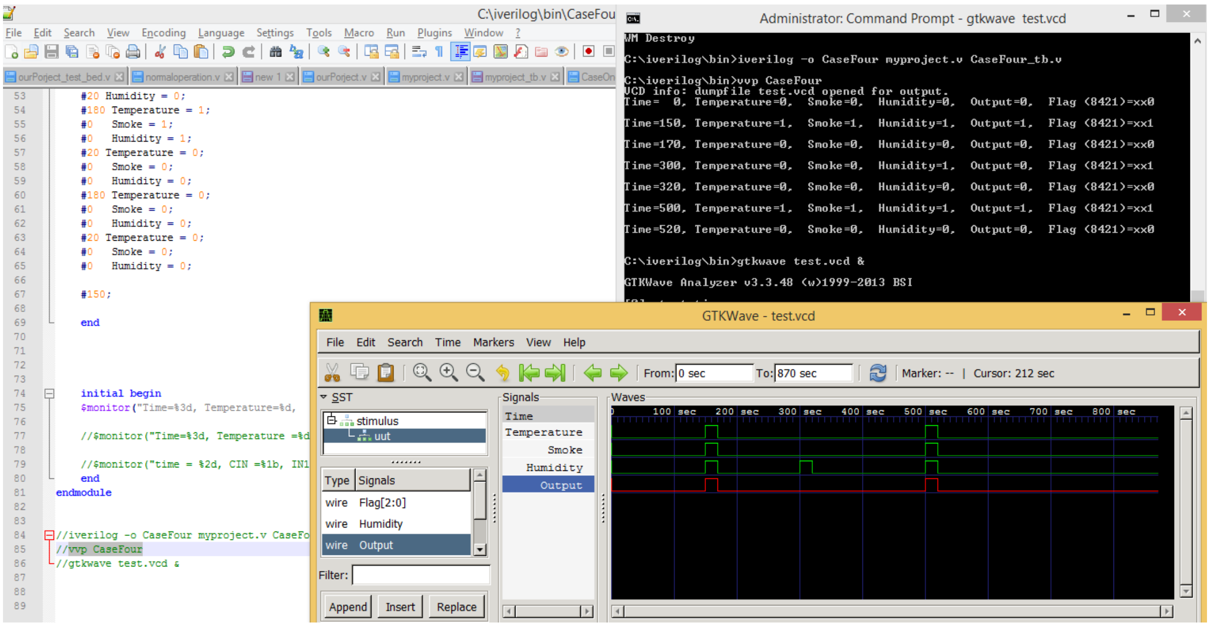

****

OurProjectSAS.V

Case 5: Figure 9 (b) GTKWave waveform for Trustworthy Event Detection using PLD/User Defined Primitive (UDP) (Logic Object Domain - I, Logic Object Domain - II, and Logic Object Domain- III) for Smart Irrigation System.

```
// OurProjectSAS.V
//Example of comparator using UDP Table for smart irrigation system (trustworthy event detevtion)
Case 5: Figure 9 (b) GTKWave waveform for Trustworthy Event Detection using PLD/User Defined Primitive (UDP) (Logic Object Domain - I, Logic Object Domain - II, and Logic Object Domain- III) for Smart Irrigation System.
```

```
`timescale 1ns / 1ps
module comparator(
        input AirTemperature,
        input SoilTemprature,
        input AirHumidity,
        input SoilMoisture,
        output Output,
        output [2:0] Flag

        );
        compare  c0(Output,  AirTemperature,  SoilTemprature,  AirHumidity, SoilMoisture);

        compare2  c20(Flag,  AirTemperature,  SoilTemprature,  AirHumidity, SoilMoisture);

        endmodule

        ///The below logic table is used for the logic gate design

        primitive compare(Output, in1, in2, in3, in4);
                output Output;
                input in1,in2,in3, in4;
        table
        // in1  in2  in3  in4: out
            0    0    0    0    :  0;
            0    0    0    1    :  0;
            0    0    1    0    :  0;
            0    0    1    1    :  1;
            0    1    0    0    :  0;
            0    1    0    1    :  1;
            0    1    1    0    :  1;
            0    1    1    1    :  1;
            1    0    0    0    :  0;
            1    0    0    1    :  1;
            1    0    1    0    :  1;
            1    0    1    1    :  1;
            1    1    0    0    :  1;
            1    1    0    1    :  1;
            1    1    1    0    :  1;
            1    1    1    1    :  1;

        endtable
        endprimitive

        /// The below logic table is used for the Flag (i.e, Gray Code), it also transfers the status of all sensors such as which sensor or variables participate in the output
```

```

primitive compare2(Flag, in1, in2, in3, in4);
        output Flag;
        input in1,in2,in3, in4;
table
// in1  in2  in3  in4: out
    0    0    0    0    :  0;
    0    0    0    1    :  1;
    0    0    1    0    :  1;
    0    0    1    1    :  1;
    0    1    0    0    :  1;
    0    1    0    1    :  1;
    0    1    1    0    :  1;
    0    1    1    1    :  1;
    1    0    0    0    :  1;
    1    0    0    1    :  1;
    1    0    1    0    :  1;
    1    0    1    1    :  1;
    1    1    0    0    :  1;
    1    1    0    1    :  1;
    1    1    1    0    :  1;
    1    1    1    1    :  1;

endtable
endprimitive
```

CaseFive_tb.v

```
//Case 5: TestBench for OurProjectSAS.v module
For trustworthy event detection (smart irrigation), we can observe three out
of four reliable events are detected at 39600, 81200, and 16400 seconds,
respectively, over 173200 seconds (48.1 Hours) of simulation time.
In this simulation, only one faulty signal is fed into the Soil Temperature
sensor, and the associated event information is captured too at 132000
Seconds.
```

```

// testbench for comparator module
`timescale 1s / 1ps
module stimulus;
        // Inputs
        reg AirTemperature;
        reg SoilTemprature;
        reg AirHumidity;
        reg SoilMoisture;
                // Outputs
                wire Output;
                wire [2:0] Flag;
```

```

    // Instantiate the Unit Under Test (UUT)
    comparator uut (
            .AirTemperature(AirTemperature),
            .SoilTemprature(SoilTemprature),
            .AirHumidity(AirHumidity),
            .SoilMoisture(SoilMoisture),
            .Output(Output),
            .Flag(Flag)
    );

    initial begin
            // Initialize Inputs
    $dumpfile("test.vcd");
$dumpvars(0,stimulus);

    AirTemperature = 0;
    SoilTemprature = 0;
    AirHumidity = 0;
    SoilMoisture = 0;
    // an event occures and all sensors observed the event
    #39600 AirTemperature = 1;
    #0  SoilTemprature = 1;
    #0  AirHumidity = 1;
    #0  SoilMoisture = 1;
    #2000 AirTemperature = 0;
    #0  SoilTemprature = 0;
    #0  AirHumidity = 0;
    #0  SoilMoisture = 0;
    // an event occures and all sensors observed the event
    #39600 AirTemperature = 1;
    #0  SoilTemprature = 1;
    #0  AirHumidity = 1;
    #0  SoilMoisture = 1;
    #2000 AirTemperature = 0;
    #0  SoilTemprature = 0;
    #0  SoilMoisture = 0;
    #0 AirHumidity = 0;
    ////////////only one sensor change due to enviroment effects
    #0 AirTemperature = 0;
    #48800  SoilTemprature = 1;
    #0  SoilMoisture = 0;
    #0 AirHumidity = 0;
    #0 AirTemperature = 0;
    #2000  SoilTemprature = 0;
    #0  SoilMoisture = 0;
    #0 AirHumidity = 0;
    // an event occures and all sensors observed the event
```

```
                #30400 AirTemperature = 1;
                #0   SoilTemprature = 1;
                #0   AirHumidity = 1;
                #0  SoilMoisture = 1;
                #2000 AirTemperature = 0;
                #0   SoilTemprature = 0;
                #0   AirHumidity = 0;
                #0  SoilMoisture = 0;
                #0 AirTemperature = 0;
                #0   SoilTemprature = 0;
                #0   AirHumidity = 0;
                #0  SoilMoisture = 0;

                #6800;

            end


                initial begin
                $monitor("Time=%3d,  AirTemperature=%d,   SoilTemprature=%d,  AirHumidity=%d,  SoilMoisture=%d,    Output=%d,    Flag  (8421)=%b\n",$time, AirTemperature, SoilTemprature, AirHumidity,  SoilMoisture, Output, Flag);

                end
        endmodule


        // C:\iverilog\bin> iverilog -o CaseFive OurProjectSAS.v CaseFive_tb.v
        // C:\iverilog\bin> vvp CaseFive
        // C:\iverilog\bin> gtkwave test.vcd &
```

Output:

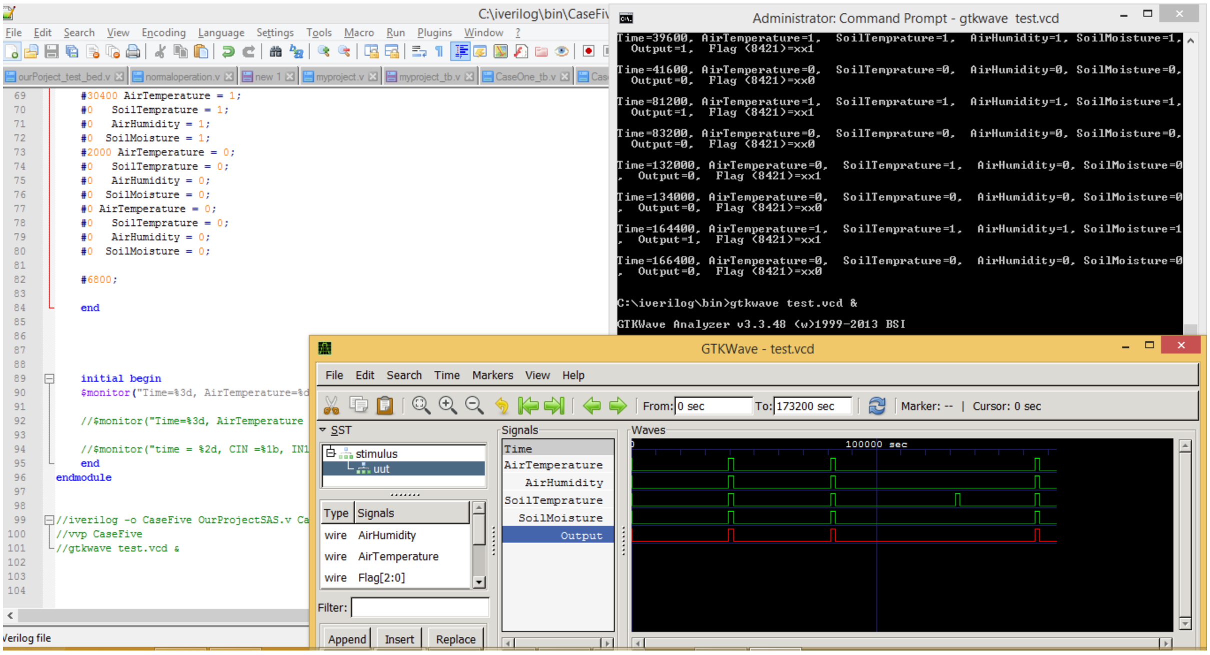

****

If you need more information or help you can contact me.

Thanks

Regards:
Hafiz ur Rahman
www.imhafiz.com
